# Systemic advantage has a meaningful relationship with grade outcomes in students’ early STEM courses at six research universities

**DOI:** 10.1186/s40594-024-00474-7

**Published:** 2024-02-23

**Authors:** Sarah D. Castle, W. Carson Byrd, Benjamin P. Koester, Meaghan I. Pearson, Emily Bonem, Natalia Caporale, Sonja Cwik, Kameryn Denaro, Stefano Fiorini, Yangqiuting Li, Chris Mead, Heather Rypkema, Ryan D. Sweeder, Montserrat B. Valdivia Medinaceli, Kyle M. Whitcomb, Sara E. Brownell, Chantal Levesque-Bristol, Marco Molinaro, Chandralekha Singh, Timothy A. McKay, Rebecca L. Matz

**Affiliations:** 1 Department of Mathematics and Statistical Science, University of Idaho, MS 1103, 875 Perimeter Dr, Moscow, ID 83844 USA; 2 Center for the Study of Higher & Postsecondary Education, University of Michigan, 2117 School of Education Building, 610 E University Ave, Ann Arbor, MI 48109 USA; 3 LSA Dean: Undergraduate Education, University of Michigan, 450 Church St, Ann Arbor, MI 48109 USA; 4 Combined Program in Education and Psychology, University of Michigan, 610 East University Ave, Suite 1400 D, Ann Arbor, MI 48109 USA; 5 Center for Instructional Excellence, Purdue University, 155 South Grant St, West Lafayette, IN 47907 USA; 6 Department of Neurobiology, Physiology and Behavior, University of California Davis, 188 Briggs Hall, Davis, CA 95616 USA; 7 Department of Physics and Astronomy, University of Pittsburgh, 3941 O’Hara St, Pittsburgh, PA 15260 USA; 8 Division of Teaching Excellence and Innovation, University of California Irvine, 3000 Anteater Instruction Research Building, 653 E Peltason Dr, Irvine, CA 92697 USA; 9 Institutional Analytics—Research and Analytics, UITS and Department of Anthropology, Indiana University Bloomington, Cyberinfrastructure Building, 2709 E 10th St, Bloomington, IN 47401 USA; 10 Department of Physics, Oregon State University, 103 SW Memorial Place, Corvallis, OR 97331 USA; 11 School of Earth and Space Exploration, Arizona State University, PO Box 871404, Tempe, AZ 85287 USA; 12 Foundational Course Initiative, Center for Research On Learning and Teaching, University of Michigan, 100 Washtenaw Ave, Ann Arbor, MI 48109 USA; 13 Lyman Briggs College and the Office of Undergraduate Education, Michigan State University, 919 E Shaw Ln, East Lansing, MI 48103 USA; 14 Program in Qualitative and Quantitative Research Methodology, Indiana University Bloomington, 201 N Rose Ave, Bloomington, IN 47405 USA; 15 Research for Inclusive STEM Education Center, Arizona State University, 427 E Tyler Mall, Tempe, AZ 85281 USA; 16 Academic Innovation and Technology/Teaching and Learning Transformation Center, University of Maryland, College Park, 4131 Campus Drive, College Park, MD 20742 USA; 17 Department of Physics, University of Michigan, 450 Church St, Ann Arbor, MI 48109 USA; 18 Center for Academic Innovation, University of Michigan, 317 Maynard St, Ann Arbor, MI 48104 USA

**Keywords:** Course grades, Generation status, Grade anomaly, Income, Introductory courses, Race/ethnicity, Sex, STEM, Systemic advantage index, Undergraduate

## Abstract

**Background:**

Large introductory lecture courses are frequently post-secondary students’ first formal interaction with science, technology, engineering, and mathematics (STEM) disciplines. Grade outcomes in these courses are often disparate across student populations, which, in turn, has implications for student retention. This study positions such disparities as a manifestation of systemic inequities along the dimensions of sex, race/ethnicity, income, and first-generation status and investigates the extent to which they are similar across peer institutions.

**Results:**

We examined grade outcomes in a selected set of early STEM courses across six large, public, research-intensive universities in the United States over ten years. In this sample of more than 200,000 STEM course enrollments, we find that course grade benefits increase significantly with the number of systemic advantages students possess at all six institutions. The observed trends in academic outcomes versus advantage are strikingly similar across universities despite the fact that we did not control for differences in grading practices, contexts, and instructor and student populations. The findings are concerning given that these courses are often students’ first post-secondary STEM experiences.

**Conclusions:**

STEM course grades are typically lower than those in other disciplines; students taking them often pay grade penalties. The systemic advantages some student groups experience are correlated with significant reductions in these grade penalties at all six institutions. The consistency of these findings across institutions and courses supports the claim that inequities in STEM education are a systemic problem, driven by factors that go beyond specific courses or individual institutions. Our work provides a basis for the exploration of contexts where inequities are exacerbated or reduced and can be used to advocate for structural change within STEM education. To cultivate more equitable learning environments, we must reckon with how pervasive structural barriers in STEM courses negatively shape the experiences of marginalized students.

**Supplementary Information:**

The online version contains supplementary material available at 10.1186/s40594-024-00474-7.

## Introduction

Despite national reform efforts dedicated to increasing diversity and inclusion in science, technology, engineering, and mathematics (STEM), these fields continue to witness a disproportionate loss of marginalized students (Seymour & Hunter, 2019). STEM education researchers have repeatedly identified unequal outcomes in traditional academic achievement measures across student groups by gender, race/ethnicity, and socioeconomic measures (Dika & D’Amico, 2016; Eddy & Brownell, 2016; Koester et al., 2016; Malespina & Singh, 2023; Matz et al., 2017; Mead et al., 2020; Whitcomb et al., 2021; Xie et al., 2015). Historically, STEM learning environments have been spaces in which systemic inequities (e.g., racism, sexism, and classism) create advantages, for example, for those who are white, wealthy, male, and continuing-generation, leading to a lack of diverse representation in STEM fields across degree programs, levels of education, and careers (Gin et al., 2022; McGee, 2020; National Center for Science & Engineering Statistics, 2021; Reinholz & Ridgway, 2021). However, most of this research has been concentrated at a sole institution or, if examined across institutions, is missing a coordinated methodology grounded in historical theoretical perspectives which position inequalities as a byproduct of university structures that perpetuate and exacerbate inequities rather than student demographics. Research on systemic, structural advantages across undergraduate STEM learning environments can uncover how these advantages persist across intersecting identities and positions. The goal of this study, then, is to explore how systemic advantages in STEM manifest across multiple large public research universities in the United States (U.S.) and highlight the need for institutions to focus on centering the experiences of students with historically marginalized identities as a step toward more equitable and inclusive learning environments for all students.

Identifying systemic inequities benefits from a comparison of student outcomes across multiple institutions using the same conceptual frameworks, methodologies, and definitions (e.g., Matz et al., 2017) as these commonly vary across studies. We argue that for a comparison to be constructive and support transformative change in STEM education, analyses must be grounded in an asset-based model using critical frameworks to examine the complex relationships between institutional practices and student identities, backgrounds, and outcomes. We use such an approach here to explore whether data from introductory STEM courses at multiple research universities support the claim that demographic-based grade differences are driven by systemic inequities. Prior to reviewing literature about systemic inequities within foundational STEM courses and their positioning as propagators of inequity, we situate what we mean by equity and how we apply critical frameworks to begin examining this variation. We then present our approach to exploring a dataset of more than 200,000 students across 60 STEM courses at 6 universities over 10 years.

## Background

### Conceptualization of equity in education

Despite our different positions, privileges, histories, and commitments as individual authors, we share a common goal of dismantling oppressive systems and pursuing equity within higher education. Before exploring related literature and theory, it is important that we define our conception of equity, specifically with respect to educational settings (Levinson et al., 2022; Russo-Tait, 2023; Wolbring & Nguyen, 2023). Drawing from Gutiérrez’s (2012) sociopolitical framework and Black feminist theory (Collins, 2000; Crenshaw, 1991; Davis, 1983; Hooks, 1981; Lorde, 1984), we view equity as acknowledgment of how historical events have created power imbalances within higher education and working to dismantle these disparities, so all students are empowered.

It follows that we do not view equity in education as a static goal (e.g., parity across grade distributions) as this approach does not guarantee equity. Rather, we position course grade disparities as a product of underlying inequitable systems and as a proxy for inequities. Two students who receive the same grade have not necessarily had equitable experiences, as fairness does not mean sameness (Gutiérrez, 2012). Indeed, the outcome of similar course grades does not necessarily redress the systems that advantage some students over others. Further, the focus on student empowerment within this conceptualization of equity is a critical dimension not addressed with the outcome of similar course grades. Students who are marginalized and minoritized often have their voices silenced, identities suppressed, and knowledge devalued. Therefore, considering identity as a precursor to power (Gutiérrez, 2012), equity focuses on addressing these power dynamics in a way that empowers students to authentically be themselves rather than conforming to inequitable and oppressive structures.

With this framing in mind, the goal is not simply to observe inequities in grades, rather it is to use this information to dismantle disparities and empower all individuals given that part of equity is grappling with how structural and political systems cause harm and produce academic disparities. In an academic context, equity could look like acknowledging how marginalized groups have had their power diminished within the classroom and academia as a whole as well as working to dismantle oppressive systems, providing resources and structures to enable empowerment. Simply put, awareness that emerges from observing inequities in grades is not the end goal; adjustment and response to power imbalances should be continuous work (Rehrey et al., 2020). Thus, equity as a process informs our theoretical approach to studying intersectional inequalities in foundational STEM courses, operationalized as large-enrollment, stable, gateway courses that serve a wide variety of students (Center for Research on Learning & Teaching, n.d.). From this conceptualization of equity, we now take a historical perspective to examine how the foundations of higher education fostered the systemic inequities that persist today.

### Historical foundation of systemic inequities

The first colleges and universities in the U.S. were made in the nation’s image (Dancy II et al., 2018; Mustaffa, 2017; Patton, 2016); like constitutional rights, access was primarily permitted to white men from wealthy Christian backgrounds (Byrd, 2021; Renn & Reason, 2021; Thelin, 2019; Wilder, 2013). Women and those who were not white or lacked wealth were denied the opportunity to pursue higher education (Renn & Reason, 2021). Many of these higher education institutions were built on land violently seized from Native Americans (Nash, 2019; Wolfe, 1999) and enslaved Black people were forced to attend to architectural developments and caretaking duties (Mustaffa, 2017; Patton, 2016; Wilder, 2013).

During the mid-1900s, U.S. colleges and universities began to significantly alter their demographic composition. At the time, social movements coincided with national pressures to make the country more globally competitive (Bell, 1980). Women and racially minoritized people had advocated throughout the 1900s on the front lines for greater access to higher education (Johnston, 2018; McCammon et al., 2001), but this was not realized until the 1950s and 1960s during the global space race. The U.S. was seeking to expand its workforce educated in STEM disciplines, thereby aligning the interests of those advocating for access to higher education with that of the majority, finally spurring action (Bell, 1980). Civil rights legislation was developed shortly after to increase access to higher education for an array of historically minoritized people including women and low-income and racially minoritized populations (Renn & Reason, 2021; Warikoo & Allen, 2020). Increasing access was not done purely for equity-oriented reasons; as a result, we still see exclusion and harm in STEM higher education contexts.

### Contemporary systemic inequities in American higher education

Although higher education admissions policy changes in the ensuing decades have substantially expanded access, colleges and universities in the U.S. remain exclusionary spaces (e.g., Dancy & Hodari, 2023). The original practice of selecting a limited few for higher education has enabled campus environments and academic structures to continue to be non-inclusive of the lived experiences of minoritized populations (Patton, 2016; Renn & Reason, 2021). For example, the valued classroom structures and discourse patterns within undergraduate mathematics have greater benefits for men than women (Johnson et al., 2020). Racially minoritized students express experiencing discrimination when navigating office hours or securing research opportunities (Masta, 2019; Tichavakunda, 2021), which negatively influences their sense of belonging and academic confidence (Duran et al., 2020; Jack, 2021; Strayhorn, 2018). Students report feeling like they must over-achieve academically in STEM environments to counteract negative stereotypes about their social group (Jack, 2021; McGee & Martin, 2011; Seymour & Hunter, 2019; Squire et al., 2018), and the psychological distress of navigating exclusionary environments has negative effects on minoritized students’ wellbeing and academic outcomes (McGee, 2021; Seymour & Hunter, 2019). Studies repeatedly show that learning environments in STEM disciplines are unwelcoming and unsupportive of students from marginalized populations, ultimately hindering their degree progression (Fiorini et al., 2023; Leyva et al., 2021; McCoy et al., 2017; McGee, 2021). Colleges and universities often fail to implement support structures that could assist minoritized students, particularly students whose secondary education falls short (Engle & Tinto, 2008; Kenyon & Reschovsky, 2014; Meatto, 2019).

Introductory STEM courses are key barriers for minoritized students (Seymour & Hunter, 2019), notable for consistently yielding grade performance differences across student populations, with women and underrepresented and racially minoritized, low-income, and first-generation students generally receiving lower grades than white, wealthy, continuing-generation men, even after accounting for students’ pre-college and family background characteristics (Dika & D’Amico, 2016; Koester et al., 2016; Malespina & Singh, 2023; Matz et al., 2017; Whitcomb et al., 2021; Wright et al., 2016; Xie et al., 2015). Inequalities at these initial stages in STEM majors hinder diversity in the STEM workforce, as lower academic performance in these courses has been shown to significantly increase the probability of students leaving STEM degree programs (King, 2015; Witteveen & Attewell, 2020). Introductory STEM courses arguably operate as key sites of intersected inequalities in higher education, and the reliance on such courses at research universities is one way that racialized, class-based, and gendered inequalities are reproduced within higher education generally, and in STEM fields particularly.

## Theoretical perspectives

### Intersectionality and organizational theory

We are guided by intersectionality and organizational theories that reveal how organizations such as colleges and universities are gendered, racialized, and classed (Acker, 1990; Armstrong et al., 2014; Bourdieu & Passeron, 1990; Byrd, 2017, 2021; Collins, 2000; Collins & Bilge, 2020; Lee, 2016; Ray, 2019).

Intersectionality describes how people’s experiences relate to multiple, intertwined social positions that reflect systems of power, including sexism, racism, and classism, influencing their everyday lives (Carbado, 2013; Cho et al., 2013; Collins, 2000; Collins & Bilge, 2020; Crenshaw, 1991). Different contexts and situations can heighten one identity compared to others even within the same spaces and places, such as moving from one classroom to another on a campus (Carbado, 2013; Collins, 2000). Marginalized identities are reified when people face structural barriers to mobility, stability, and success, and when combined with their other identities, existing at a particular intersection can yield qualitatively different experiences. The concept of intersectionality helps researchers grapple with the complexity of a person’s experiences and outcomes within an unequal opportunity structure (Bowleg, 2008; Collins & Bilge, 2020; Crenshaw, 1991). Within higher education, it is important to understand opportunity structures and the ways that the purposes and goals of organizations bring about differential resources and opportunities for students to pursue along their degree pathways.

It is also important to avoid oversimplifying intersectionality, placing inequalities within social identities rather than reflecting unequal opportunity structures (Carbado & Harris, 2019; Cho et al., 2013; Collins & Bilge, 2020; Harris & Patton, 2018; Haynes et al., 2020). Decoupling a person’s experiences and outcomes from interlocking systems of power individualizes inequalities, leaving unaddressed organizational features that perpetuate inequalities long-documented in higher education and particularly in STEM fields (Allen & Jewell, 2002; Armstrong & Jovanovic, 2017; Bauer et al., 2021; Brunn-Bevel et al., 2019; Byrd, 2021; Fiorini et al., 2023; Griffin, 2019; Thelin, 2019). Further, this disconnect can misinform initiatives and policies aiming to address such inequities by missing how organizations differentially distribute resources and opportunities through policies, practices, and everyday interactions (Acker, 1990; Byrd, 2021; Ray, 2019).

Persistent inequities across STEM fields hamper the capacity to uphold institutional missions and ideals of supporting students with an array of different identities and from different backgrounds. Although STEM disciplines notably show greater inequities compared to other fields of study (Matz et al., 2017; Riegle-Crumb et al., 2019), research continues to find that systemic inequities are a common feature of higher education across academic fields, levels of degree programs, and employment (Blair-Loy & Cech, 2022; Byrd, 2021; Byrd et al., 2019; Espinosa et al., 2019; McGee, 2020; Posselt, 2018, 2020; Stewart & Valian, 2018; Zambrana, 2018). Therefore, we must be mindful in interpreting research findings to avoid deficit framing based on presumptions of meritocracy that can limit interventions and policy changes by focusing on individuals and not universities (Blair-Loy & Cech, 2022; Carnevale et al., 2020; Castillo & Gillborn, 2022; Harper, 2010). Given that inequities are so intertwined with higher education, and STEM fields in particular, a theoretical framework that situates how individual academic performance reflects organizational inequity is warranted.

### Intersectionality-informed explorations of quantitative STEM education data

The application of intersectionality alone to quantitative studies can further disconnect inequities and inequalities from the contexts surrounding people if not paired with a critical lens that seeks to emphasize the societal context of a phenomenon (Pearson et al., 2022). Therefore, we couple the guidance of intersectional and organizational theories with a critical approach to raise questions about the orientation to and purposes of quantitative methods for understanding marginalization within higher education. This disposition helps to (1) avoid assumptions about the value neutrality of quantitative analyses; (2) limit deficit perspectives of individuals and groups that essentialize people; (3) support consideration of the contexts that lead to particular outcomes; (4) reconsider sociodemographic groupings and categories; and (5) recognize that interpretations of quantitative data are privileged in society. We explicitly highlight these dispositional tenants throughout the paper to show where they guided decisions and interpretations. As researchers, we must carefully consider how we assign meaning to interpretations of data that can silence the voices and experiences of marginalized communities as data do not speak for themselves (Byrd, 2021; Castillo & Gillborn, 2022; Covarrubias & Vélez, 2013; D’Ignazio & Klein, 2020; Garcia et al., 2018; Gillborn et al., 2018; López, et al., 2018; Pearson et al., 2022).

While tending to each of these critical quantitative tenets can enhance research to speak to the systemic realities of inequities in STEM learning environments, lingering issues must be addressed when applying intersectionality to quantitative data. Researchers often use individual-level variables without attending to historical context or the systems in which individuals operate, thereby hindering the visibility of intersected experiences, processes, and outcomes of systemic phenomena (Bauer et al., 2021; Bowleg, 2008; Hancock, 2007, 2013; Lopez et al., 2018). It is important to note how such exploration is constrained by the variables used in analyses. For example, the ways that institutions and researchers place students in sociodemographic categories by gender, race/ethnicity, and socioeconomic position (i.e., income and first-generation status) are not always documented (Byrd, 2021), nor can we assume identity processes of students (i.e., how someone self-identifies and why). In this study, we explicitly attempt to link our analysis to such historical context.

In relation to interpretations of sociodemographic variables, this framing means that a coefficient for students identifying with a particular racial/ethnic group in a multivariate model should not be interpreted by researchers as the social construct of race directly influencing a student’s outcome such as grades (Bonilla-Silva, 2006). Rather, a racial disparity in course grades reflects the racially inequitable learning contexts of that course that impact students along racial lines. Through additive approaches, researchers might explore the average combined effects of multiple systems of inequity and power, but also should recognize that, of course, qualitatively different experiences underlie overarching quantitative patterns and are not fully captured. Grounded in these theoretical perspectives, our approach interrogates the entrenched nature of systemic oppression in postsecondary STEM courses.

## Research questions

Our critical theoretical perspectives, informed by intersectionality and organizational theory, provide the foundation for this study about how introductory STEM courses operate as key sites of intersected inequalities in higher education. We investigate grade disparities as a reflection of existing institutional practices and policies that perpetuate inequalities among students by sex, race/ethnicity, and socioeconomic status. To explore such systemic inequalities and expand our scope of cross-institutional analyses beyond prior work focused solely on sex (Matz et al., 2017), we define and use the systemic advantage index (SAI), which represents the total number of advantages that characterize students within institutions according to sex, race/ethnicity, income, and first-generation status. To explore the extent to which historically based (dis)advantages exist across institutions and to document the manifestation of systemic inequities in introductory STEM courses, we ask the following research questions:

1. What is the distribution of students by systemic advantage at each institution?
2. What is the relationship between systemic advantage and course outcomes?

## Methods

### Institutional context

This study examines data from six four-year public universities in the U.S.: the flagship campuses of Arizona State University, Indiana University, Michigan State University, Purdue University, the University of Michigan, and the University of Pittsburgh, randomly deidentified herein as Institutions A through F. These six institutions are similar in that they are doctoral universities with very high research activity, they have large enrollments (from 19,000 to 42,000 undergraduate students in Fall 2018; National Center for Education Statistics, n.d.), and they serve populations that are primarily residential (Carnegie Classification of Institutions of Higher Education, n.d.).

Other key characteristics vary; for example, in 2018–2019, the admissions rate ranged from 23 to 85% across the institutions and the 6-year graduation rate ranged from 63 to 93% (National Center for Education Statistics, n.d.). The percentages of undergraduate students who are women (42 to 52%) and non-white (30% to 50%) also vary (Fall 2018 figures; National Center for Education Statistics, n.d.). The data used in this study were gathered and derived from the student information systems maintained at each institution following Institutional Review Board requirements. The student-level data were held locally, not shared between researchers.

### Data collection

#### Defining the sample: student population (step 1)

**Fig. 1:**
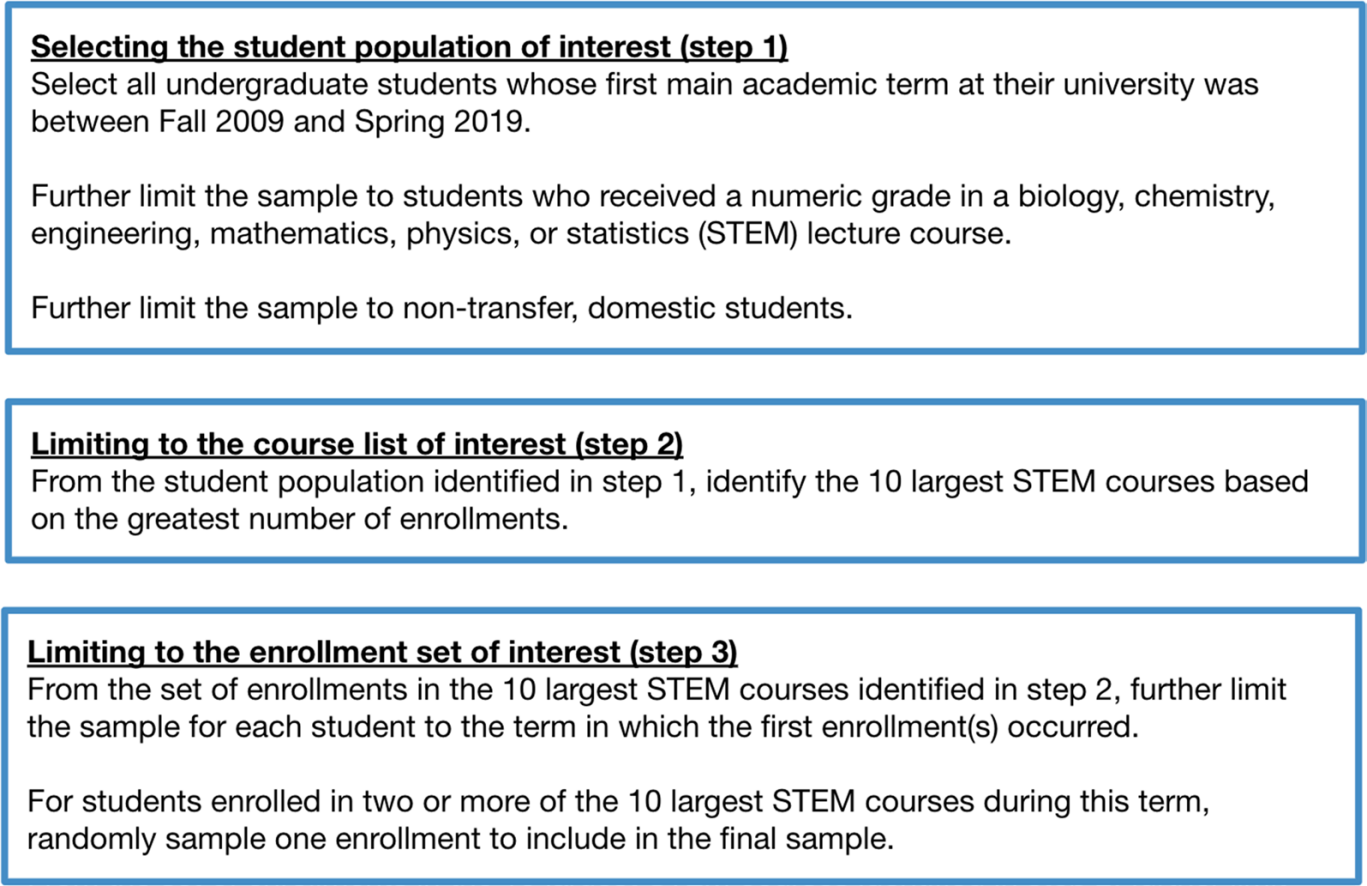
Summary of criteria and exclusions for the data sample

We selected all undergraduate students who received a numeric grade in at least one STEM course (defined as those with biology, chemistry, engineering, mathematics, physics, or statistics course codes, excluding laboratory courses as they tend to be secondary to lecture courses) within their first academic year at one of the participating universities spanning a period of 10 academic years (Fall 2009 through Spring 2019, excluding summer terms as we were interested in students’ first interactions with STEM courses). All data were thus collected before the instructional, grading, and policy changes that resulted from the COVID-19 pandemic beginning in March 2020. A summary of the criteria and exclusions applied to the sample in this and the following steps is shown in Fig. 1.

We excluded transfer students as our goal was to examine students’ first interactions with post-secondary STEM courses. Though a subset of transfer students did take their first STEM course at one of our institutions and could have been included in principle, the transfer credit records we accessed were not consistently specific enough at the course level to parse these groups; we assumed that some transfer students had prior STEM courses transfer only as general education or elective credit, and thus that we would not be able to reliably determine if a student had or had not taken a post-secondary STEM course before transferring. International students were also excluded because race is a social construct defined differently according to each country’s history and culture. Given that our data came from entirely U.S.-based institutions, we did not want to enforce a potentially inappropriate U.S.-based construct on our international student populations. With these sample limitations, we do not assert that transfer and international students do not experience inequitable learning environments. Rather, we aimed to explore different axes of systemic advantage here.

#### Defining the sample: the largest STEM courses (step 2)

For this study, we defined the 10 largest STEM courses at each university as the courses with the greatest number of enrollments from this student population across the 10-year period of interest. We included courses without regard to level; in particular, the number and type of mathematics courses offered prior to calculus differs across these institutions. In this way, we focused our attention on the largest STEM courses unique to each institution and its student population. The distribution of courses by discipline is shown in Table 1. Mathematics courses are the most prominent, comprising more than half of the courses overall. No upper-division courses, that is, those at the “300-level” or above, were evident in the sample (though disparities have been shown to persist at the upper-division level; see Farrar et al., 2023).

**Table 1:** The distribution of the 10 largest STEM courses at each institution by discipline

Discipline	A	B	C	D	E	F	Total
Biology	1	3	1	1	1	1	8
Chemistry	2	3	2	1	2	2	12
Engineering	0	0	0	0	0	1	1
Mathematics	6	4	6	7	5	4	32
Physics	0	0	1	1	1	1	4
Statistics	1	0	0	0	1	1	3
Total	10	10	10	10	10	10	60

#### Defining the sample: enrollment exclusions (step 3)

Two final adjustments to the sample were made. First, the enrollment patterns for students across these 10 largest courses varied; that is, each student fell into one of three categories: (1) the student took one or more of the 10 largest STEM courses in the fall term and none in the spring term; (2) the student took one or more of the 10 largest STEM courses in the spring term and none in the fall term (for example, some students began their undergraduate careers in the spring); or (3) the student took one or more of the 10 largest STEM courses in both the fall and spring terms. Because we intended to focus on students’ first experience with large STEM courses, we limited our enrollments of interest for those in group 3—the “both fall and spring” students—to only those that occurred in the students’ fall term.

Second, we adjusted the sample in the cases where students were enrolled in two or more of the 10 largest STEM courses within the same term. To support the robustness of the analyses, among the students that were represented with multiple enrollments across these 10 largest STEM courses within the same term, we randomly sampled a single STEM course enrollment for each student. This step was necessary because the GPAOs (defined below) for these course enrollments for each student would be highly correlated. This exclusion had the effect of reducing the sample of 386,035 enrollments across the 10 largest STEM courses to 227,413 enrollments. In this way, the final sample [*n* = 227,413 students (Institution A: 59,412, B: 43,287, C: 22,056, D: 34,967, E: 47,373, F: 20,318); 60 courses; 6 institutions] includes only one unique enrollment per student.

#### Demographic variables

We used four demographic variables in this study: sex, race/ethnicity, income, and first- versus continuing-generation status.

Though gender, not sex, was our construct of interest with respect to systemic advantage in undergraduate STEM courses, gender data were not available in the student records at every institution, a byproduct of the non-neutrality of data as noted in dispositional tenant 1. Thus, we used a binary sex classification (female students and male students) and recognize these data as limiting (D’Ignazio & Klein, 2020), particularly with respect to non-binary and genderqueer students.

Race/ethnicity was coded as white or non-white, where non-white included students who indicated they were American Indian or Alaska Native, Asian or Asian American, Black or African American, Hispanic, Native Hawaiian or Other Pacific Islander, or two or more races. We recognize that the choice to group students of non-white races and ethnicities into one category has the potential to perpetuate a centering of whiteness and encourage the monolithic view of non-white individuals, an implication with which we disagree. This choice was based on history, that is, U.S. universities being designed solely for white students and implementing exclusionary practices for non-white students. Our binary coding of race and ethnicity is intended to reflect this historical structural perspective.

Low-income students were defined as those eligible for federal Pell grants; however, at two institutions, these data were unavailable to researchers. In these cases, the low-income category was defined by the median income level associated with students’ high school zip code based on data from the U.S. Census Bureau; zip code has been found in prior research to be a reasonable proxy for socioeconomic status (Berkowitz et al., 2015; Link-Gelles et al., 2016). We used a median income of $46,435 or less as a conservative estimate for low income as this income level is twice the average federal poverty guideline for a family of four persons within the U.S. over our period of interest (Office of the Assistant Secretary for Planning and Evaluation, n.d.).

First-generation students were defined as those reporting that no parent or guardian had earned a bachelor’s degree.

When students were missing information for any demographic variable, they were categorized with the advantaged group in order to be conservative with the analyses.

#### Course grades and metrics

We collected students' final course grades, excluding non-numeric grades like lapsed incomplete grades, grades earned under pass/fail policies, and grades representing course or term withdrawals. The numeric grading scale was the same across three institutions, while the other three institutions’ scales each varied slightly (Table 2). Though these variations certainly impact the precise magnitude of our estimates (indeed, grading schemes have been shown to vary even across sections of the same course within a single institution; James, 2023), we maintain that the overall trends and resulting interpretations are insensitive to them.

**Table 2:** The numeric grading scales represented across the six institutions

Grade	Grading scale^a^			
	1	2	3	4
A +	4.00	4.33	4.00	4.00
A	4.00	4.00	4.00	4.00
A−	3.70	3.67	3.50	3.75
B +	3.30	3.33	3.50	3.25
B	3.00	3.00	3.00	3.00
B−	2.70	2.67	2.50	2.75
C +	2.30	2.33	2.50	2.25
C	2.00	2.00	2.00	2.00
C−	1.70	–^b^	1.50	1.75
D +	1.30	–	1.50	1.25
D	1.00	1.00	1.00	1.00
D−	0.70	–	–	0.75
E/F	0.00	0.00	0.00	0.00

^a^Grading scale 1 is used at three institutions in the sample. Grading scales 2, 3, and 4 are each used by one institution

^b^The ‘–’ notation indicates that this grade is not available at this institution

We used grade point average in other courses (GPAO) as a control metric for academic performance, defined as a student’s cumulative GPA across all courses (including non-STEM courses) and all terms excluding only the STEM course of interest (Huberth et al., 2015; Koester et al., 2016). For example, if a student enrolled in five courses during their second term, their GPAO for one of these second-term courses is calculated as their average GPA across the other four courses from that term plus all their courses from the first term. That is, GPAO is a way of describing the grades that students typically earn across all their courses while retaining the ability to compare to a student’s grade in a particular course of interest.

Because GPAO is calculated relative to each course enrollment, a student’s GPAO for one course can be different from their GPAO for another course. We selected GPAO as a control metric for academic performance because prior studies have shown its power in predicting academic outcomes over and above high school GPA and standardized exam scores (Huberth et al., 2015; Koester et al., 2016) and in highlighting inequities in STEM courses (Matz et al., 2017). Using GPAO also facilitates cross-institutional studies because it easily accounts for the variance in grading across universities; indeed, four grading scales are represented here among only six universities. With GPAO, grade comparisons are made relative to how students usually perform at their specific institution.

### Analytical framework

We calculated a metric that we call the systemic advantage index (SAI) (Castle et al., 2021) for each student based on selected demographic information as a measure that partially represents systemic oppression within the U.S. higher education system. The SAI is derived from the historical academic structures of inequity within this system and is therefore intended to be a structural measure rather than a deficit-oriented measure, aligning with dispositional tenants 2, 3, and 4.

We define SAI as the number of advantages that a student has based on their sex, race/ethnicity, income, and first- versus continuing-generation status, where male students, white students, higher-income students, and continuing-generation students, respectively, are considered advantaged. Though privilege in higher education operates through other dimensions (e.g., ableism; Reinholz & Ridgway, 2021), we limited the current study to these four characteristics based on data availability and consistency. Herein, students range from having zero advantages (i.e., first-generation, low-income, non-white female students) to four advantages (i.e., continuing-generation, higher-income, white male students).

Within this range, there are 16 mutually exclusive groups of students (Table 3); students with the same SAI have the same number of systemic advantages, but the precise advantages can differ. While each advantage contributes equally to a student’s SAI, we acknowledge that the advantages are not necessarily equivalent in how they relate to the outcomes in a course, a key limitation in line with dispositional tenant three. The same index value represents different intersectional combinations of student characteristics and, hence, conflates different experiences. However, the SAI as a coarse estimate facilitates analysis of how these axes of advantage manifest systematically within students’ introductory STEM course outcomes across institutions, providing an advantage over studies of single institutions and single axes of diversity and even meta-analyses that entail methodological variation. That is, our focus is on the system of advantages inherited and persisting in our educational systems, not on individual students or advantages, nor on how students with specific combinations of characteristics experience systemic inequities.

**Table 3:** The percentage of students by systemic advantage index (SAI) within each institution

SAI	Description	A	B	C	D	E	F
0	First-generation, lower-income, non-white female students	6	3	5	1	< 1	1
1	First-generation, lower-income, white female students First-generation, lower-income, non-white male students First-generation, higher-income, non-white female students Continuing-generation, lower-income, non-white female students	13	8	10	5	4	6
2	First-generation, lower-income, white male students First-generation, higher-income, white female students Continuing-generation, lower-income, white female students First-generation, higher-income, non-white male students Continuing-generation, lower-income, non-white male students Continuing-generation, higher-income, non-white female students	24	17	18	22	18	19
3	First-generation, higher-income, white male students Continuing-generation, lower-income, white male students Continuing-generation, higher-income, white female students Continuing-generation, higher-income, non-white male students	35	40	39	43	45	46
4	Continuing-generation, higher-income, white male students	22	33	28	29	33	29

*Note:* As an example, 6% of students at Institution A had an SAI of 0

Our analysis follows Matz et al. (2017), using students’ grades and their grades in other courses (their GPAO) together to calculate a metric called grade anomaly. Grade anomaly is the difference between course grade and GPAO. A positive grade anomaly indicates that the student received a higher grade in the sampled course compared to their other courses—a grade “bonus”. A negative grade anomaly indicates that the student received a lower grade in the sampled course compared to their other courses—a grade “penalty”. This simple comparison is easy to compute, widely available, and informative both as a kind of control for academic performance at the university level and in providing a measure of the feedback to the student about how well they did in their course compared to their typical performance. We used ordinary least squares regression to evaluate the relationship between SAI and grade anomaly. All analyses and visualizations were carried out using R Statistical Software (R Core Team, 2023).

## Results

### RQ1: What is the distribution of students by systemic advantage at each institution?

**Fig. 2:**
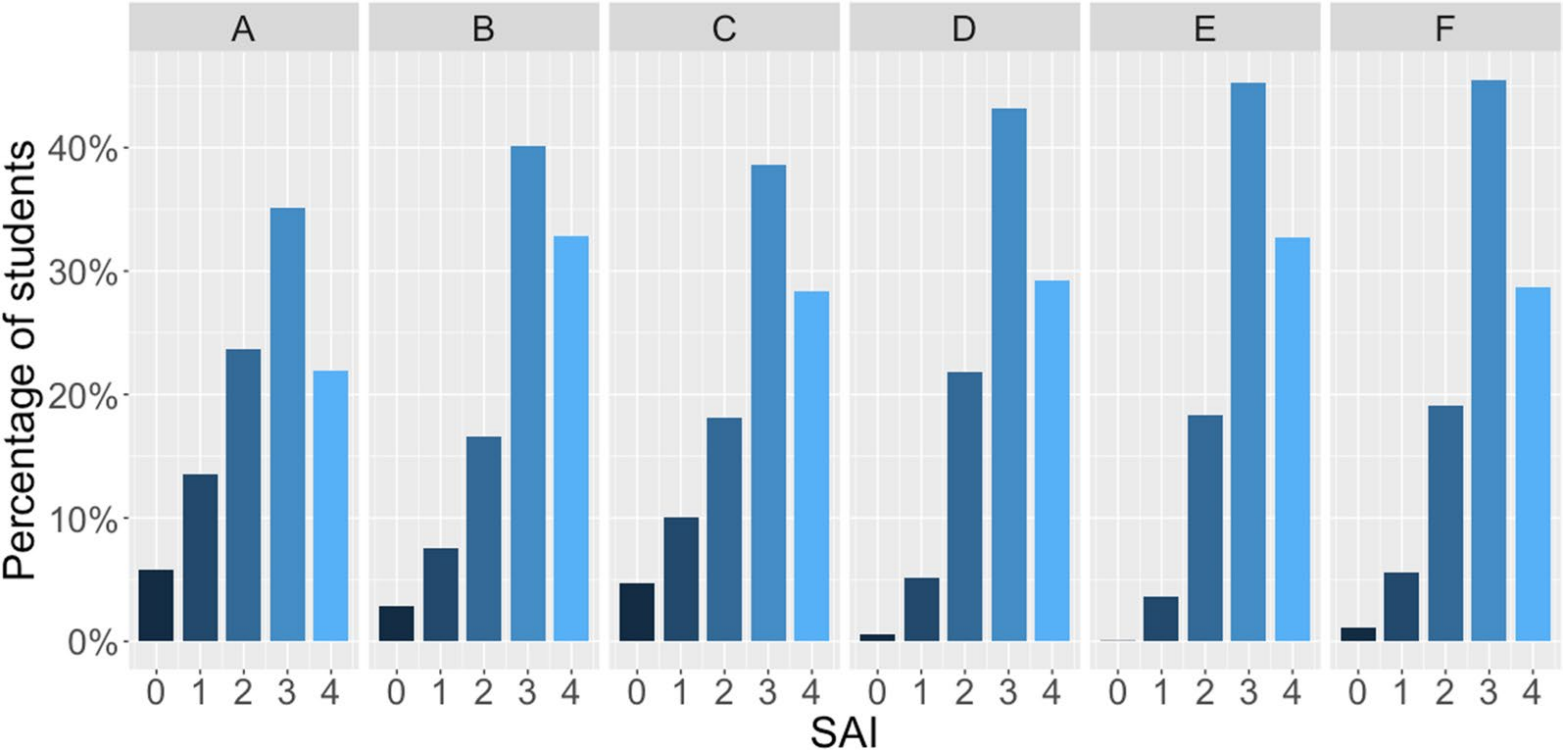
The percentage of students by systemic advantage index (SAI) within each institution

We first evaluated the percentage of students in each SAI group by institution (Table 3; Fig. 2) to understand the distribution of students by SAI groups and to explore similarities and differences in the population at each university. At every institution, the majority of students were represented by groups with three or four advantages. Institution A had the smallest proportion of students (57%) with three or four advantages; though not a statistical outlier, it was furthest from the other institutions. Institutions B, D, E, and F all had greater than 70% of students in groups with three or four advantages and, in particular, Institution E had very few students (< 1%) in SAI group 0. Institution A, and Institution C to a lesser extent, showed a broader distribution over the five SAI groups in general in comparison to the other institutions.

**Fig. 3:**
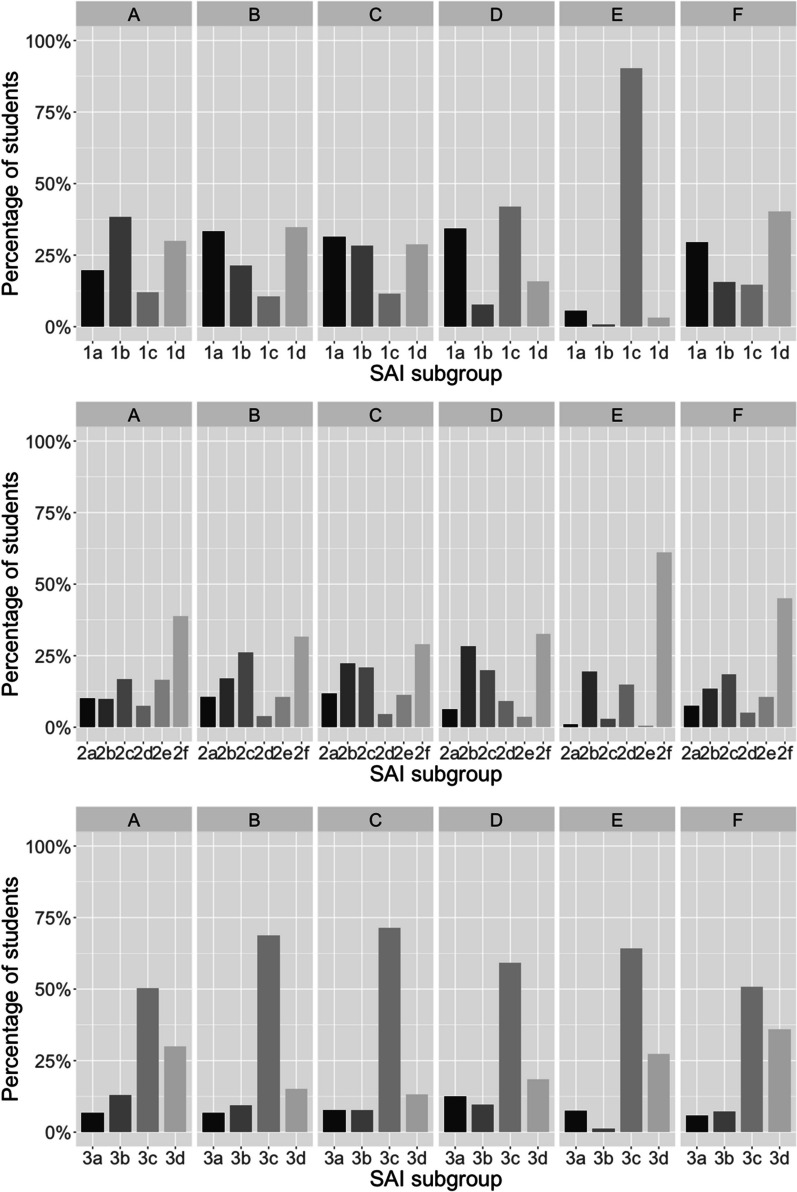
The percentage of students by systemic advantage index (SAI) subgroup with SAI = 1 (top), SAI = 2 (middle), or SAI = 3 (bottom) at each institution; see Table 4 for the description of each subgroup

The percentages of students within each SAI subgroup—for example, the percentage of first-generation, lower-income, white female students relative to the three other SAI = 1 subgroups—are provided in Table 4 and Fig. 3. Across the institutions, the SAI = 3 group is dominated by continuing-generation, higher-income, white female students and secondarily by continuing-generation, higher-income, non-white male students. The SAI = 2 group is dominated by continuing-generation, higher-income, non-white female students whereas the SAI = 1 subgroups are more evenly distributed. Considering all these data, an overall lack of low-income students is especially apparent at Institution E.

**Table 4:** The percentage of students by systemic advantage index (SAI) subgroup within each institution and SAI

SAI/SAI subgroup	Description	A	B	C	D	E	F
0	First-generation, lower-income, non-white female students	100	100	100	100	100	100
1a	First-generation, lower-income, white female students	20	33	31	34	5	29
1b	First-generation, lower-income, non-white male students	38	21	28	8	1	16
1c	First-generation, higher-income, non-white female students	12	10	11	42	90	15
1d	Continuing-generation, lower-income, non-white female students	30	35	29	16	3	40
2a	First-generation, lower-income, white male students	10	11	12	6	1	8
2b	First-generation, higher-income, white female students	10	17	22	28	20	13
2c	Continuing-generation, lower-income, white female students	17	26	21	20	3	18
2d	First-generation, higher-income, non-white male students	8	4	5	9	15	5
2e	Continuing-generation, lower-income, non-white male students	17	11	11	4	< 1	11
2f	Continuing-generation, higher-income, non-white female students	39	32	29	33	61	45
3a	First-generation, higher-income, white male students	7	7	8	13	7	6
3b	Continuing-generation, lower-income, white male students	13	9	8	10	1	7
3c	Continuing-generation, higher-income, white female students	50	69	71	59	64	51
3d	Continuing-generation, higher-income, non-white male students	30	15	13	18	27	36
4	Continuing-generation, higher-income, white male students	100	100	100	100	100	100

As an example, 20% of students in the SAI = 1 group at Institution A are first-generation, lower-income, white female students

### RQ2: What is the relationship between systemic advantage and course outcomes?

**Fig. 4:**
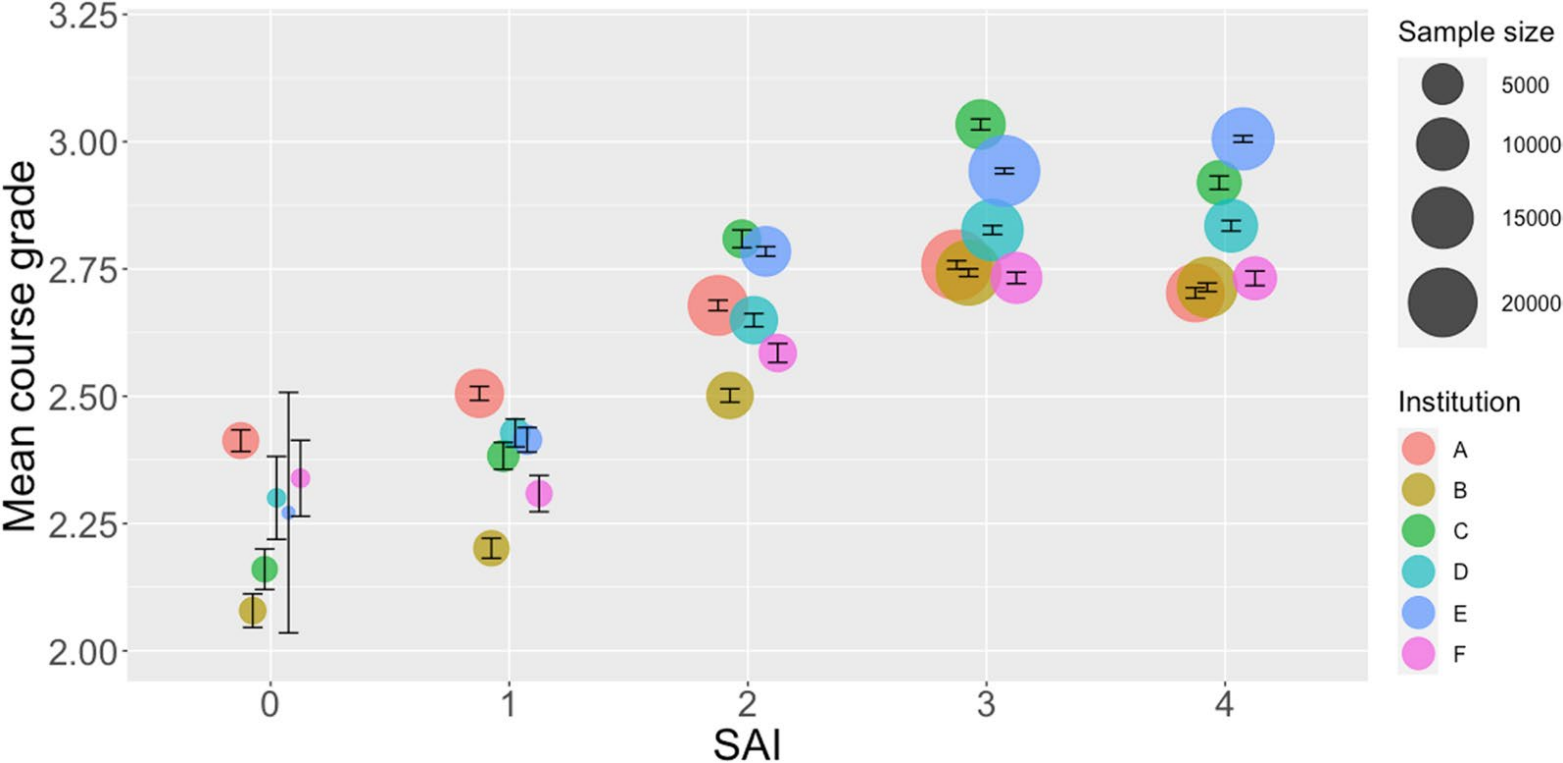
Students’ mean course grade ± 1 standard error in the sampled STEM course by systemic advantage index (SAI)

**Fig. 5:**
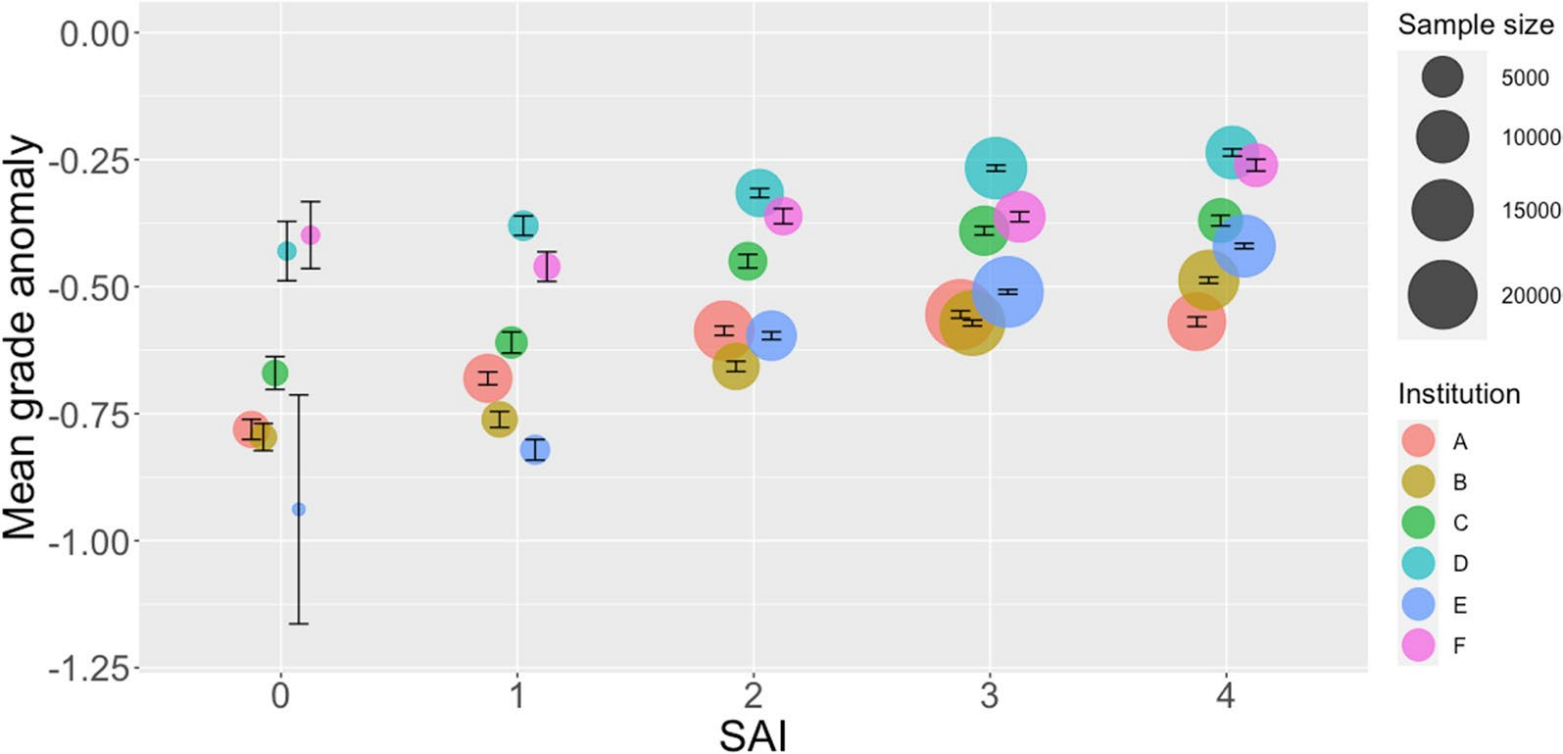
Students’ mean grade anomaly ± 1 standard error in the sampled STEM course by systemic advantage index (SAI)

We then explored the relationship between systemic advantage and two academic performance variables in the sampled STEM courses at each institution: course grade (Table 5; Fig. 4) and grade anomaly (Table 6; Fig. 5). At every institution, both relationships showed that more favorable course outcomes were generally associated with more systemic advantages. The trends were strikingly similar across universities even though we did not control for differences in grading practices, contexts, and instructor and student populations. One point of contrast is that for all but one institution (Institution E), the average course grade for SAI group 4 was the same or less than that for SAI group 3. However, when considered relative to students’ performance in other courses (Fig. 5), the trend of more favorable course outcomes for those with more advantages was still apparent.

**Table 5:** Mean course grade for each systemic advantage index group (0 to 4) by institution (A to F)

	0			1			2			3				4		
	*N*	*M*	*SD*	*N*	*M*	*SD*	*N*	*M*	*SD*	*N*	*M*	*SD*	*N*		*M*	*SD*
A	3455	2.41	1.25	8018	2.51	1.23	14,095	2.68	1.21	20,845	2.76	1.19	12,999		2.70	1.18
B	1220	2.08	1.15	3259	2.20	1.12	7198	2.50	1.12	17,379	2.74	1.00	14,231		2.71	1.00
C	1045	2.16	1.28	2225	2.38	1.25	4004	2.81	1.10	8526	3.03	0.99	6256		2.92	1.04
D	212	2.30	1.19	1798	2.43	1.16	7623	2.65	1.13	15,105	2.83	1.08	10,229		2.83	1.05
E	21	2.27	1.08	1706	2.41	1.00	8687	2.78	0.87	21,461	2.94	0.80	15,498		3.01	0.80
F	222	2.34	1.11	1139	2.31	1.20	3873	2.59	1.16	9248	2.73	1.09	5836		2.73	1.09

**Table 6:** Mean grade anomaly for each systemic advantage index group (0 to 4) by institution (A to F)

	0			1			2			3			4		
	*N*	*M*	*SD*	*N*	*M*	*SD*	*N*	*M*	*SD*	*N*	*M*	*SD*	*N*	*M*	*SD*
A	3455	− 0.78	1.16	8018	− 0.68	1.14	14,095	− 0.59	1.08	20,845	− 0.55	1.06	12,999	− 0.57	1.08
B	1220	− 0.80	0.93	3259	− 0.76	0.89	7198	− 0.66	0.85	17,379	− 0.57	0.77	14,231	− 0.49	0.73
C	1045	− 0.67	1.04	2225	− 0.61	0.98	4004	− 0.45	0.85	8526	− 0.39	0.78	6256	− 0.37	0.82
D	212	− 0.43	0.85	1798	− 0.38	0.82	7623	− 0.32	0.78	15,105	− 0.27	0.74	10,229	− 0.24	0.72
E	21	− 0.94	1.03	1706	− 0.82	0.84	8687	− 0.60	0.71	21,461	− 0.51	0.66	15,498	− 0.42	0.66
F	222	− 0.40	0.98	1139	− 0.46	0.98	3873	− 0.36	0.93	9248	− 0.36	0.95	5836	− 0.26	0.89

In particular, regression models (Table 7) showed that increasing SAI had a significantly positive relationship with grade anomaly; a one-unit increase in SAI was associated with an increase in grade anomaly of between 0.04 and 0.10 points, depending on the institution, on average (see the Estimate column in Table 7; the lowest estimate is 0.04 for Institution D and the highest estimate is 0.10 for Institution E). Further, grade anomaly in these early STEM courses was significantly greater for the most advantaged group of students (SAI = 4) versus all other students at every institution (Table 8; differences in grade anomaly for all possible comparisons of SAI groups are provided in Additional file 1).

**Table 7:** Estimates, standard errors of the mean (SEM), and t values from generalized linear regression models for the effect of systemic advantage index (SAI) on grade anomaly

Institution	Covariate	Estimate	*SEM*	*t*
A	Intercept	− 0.710	0.011	− 65.55
	SAI	0.045	0.004	11.61
B	Intercept	− 0.828	0.011	− 72.14
	SAI	0.085	0.004	23.00
C	Intercept	− 0.635	0.015	− 41.98
	SAI	0.073	0.005	14.43
D	Intercept	− 0.405	0.014	− 28.87
	SAI	0.044	0.005	9.67
E	Intercept	− 0.828	0.012	− 67.93
	SAI	0.104	0.004	27.15
F	Intercept	− 0.495	0.023	− 21.99
	SAI	0.053	0.007	7.25

The model was run separately at each institution because of restrictions on sharing student-level data across universities

*p* < 0.001 for all covariates

**Table 8:** Welch’s two-sample *t*-test for differences in grade anomaly for SAI = 0, 1, 2, or 3 versus SAI = 4 at each institution

Institution	Grade anomaly (*M*)		*t*	*df*	95% CI		*p*
	SAI 0, 1, 2, or 3	SAI 4			*LL*	*UL*	
A	− 0.60	− 0.57	3.18	21,006	0.013	0.055	0.001
B	− 0.62	− 0.49	17.46	31,042	0.121	0.151	< 0.001
C	− 0.45	− 0.37	6.48	11,896	0.056	0.104	< 0.001
D	− 0.29	− 0.24	5.96	19,669	0.035	0.068	< 0.001
E	− 0.55	− 0.42	20.02	32,064	0.118	0.144	< 0.001
F	− 0.37	− 0.26	7.81	11,431	0.082	0.137	< 0.001

We note that across all SAI groups and institutions, the average grade anomaly for students in their first STEM course, as we have defined them here, was negative. All SAI groups on average received a grade penalty—a lower grade in the sampled course compared to their other courses—ranging between 0.24 and 0.94 grade points (see the Mean columns in Table 6; the lowest mean is − 0.94 for SAI group 0 at Institution E and the highest mean is − 0.24 for SAI group 4 at Institution D), but the penalties were more amplified on average for students in SAI groups with fewer systemic advantages.

## Discussion

This study uses a simple method, grounded in a critical historical perspective, to highlight how early university STEM courses provide more favorable course outcomes to students with more systemic advantages, sustaining and increasing disparities between different student populations. Though we found that all students received lower course grades on average in introductory STEM courses relative to their other courses, the most disadvantaged groups of students, as defined by the number of disadvantages, received the largest penalties. The relationship between greater advantage and less grade penalty was significant at each institution, resonating across the broad set of disciplines and contexts represented in students’ first post-secondary interactions with STEM. Although some might argue that the grade anomaly differences result more from student effort than from the learning environment, using GPAO as a control for academic performance mitigates this alternative explanation as it accounts for students’ general study habits across a range of other courses. Further, we contend that these analyses are conservative because withdrawals were excluded, and withdrawals contribute substantially to differences in course outcomes between student groups based on gender, race/ethnicity, income, and first- versus continuing-generation status (Michaels & Milner, 2021). Withdrawals represent another source of information for examining inequities in future work.

Mathematics accounted for approximately half of the courses in the sample. Unlike other disciplines, mathematics courses are often pre- and co-requisite for many STEM degree pathways and are often a general education requirement. Therefore, the trend across institutions of more favorable introductory STEM course outcomes for those with a greater number of systemic advantages is highly concerning. An unfavorable grade in an initial mathematics course can bar students from other discipline-specific courses, forcing them to potentially extend the time to completion of their degree, or spurring them to exit their STEM program. When considering enacting structural change across institutions, it is important to note that STEM itself is not a monolith and the variations in disciplinary cultures are essential to consider when advocating for structural reform across departmental and institutional levels (Reinholz et al., 2019). Therefore, this study adds to the call for a larger conversation about undergraduate mathematics education (Reinholz et al., 2020), with a specific emphasis on the earlier mathematics courses, that centers on structures and challenges deficit discourse (Adiredtja & Louie, 2020). It is critical that equity practices promote a reconfiguration of university mathematics practices, and that researchers and educators grapple with the institutional factors that can hinder change (Ching & Roberts, 2022). As this pattern was not confined to a sole institution, it is important for future work to consider the enactment of disciplines within the university context and examine the discipline through a lens focused on systemic inequity in addition to the course- and institution-level analyses.

Initiatives both national and local to our universities have promoted diversity within STEM disciplines specifically regarding retention of students typically disadvantaged by higher education (Asai, 2020). But given that early STEM course grades are a key factor in STEM retention (Byars-Winston et al., 2010; Dika & D’Amico, 2016; King, 2015; Seymour & Hunter, 2019; Stinebrickner & Stinebrickner, 2014; Witteveen & Attewell, 2020), our findings are concerning and suggest that continued scrutiny for structural inequities in STEM is necessary. Indeed, higher grades in beginning STEM courses, especially relative to other courses, is a predictor of retention (Griffith, 2010), though patterns in persistence differ by demographic characteristics (Costello et al., 2023). Past research provides mechanisms for how inequities in early STEM courses can reflect unequal learning environments rather than student abilities. For example, Black students face implicit and explicit messages that they do not belong in STEM spaces (Basile & Black, 2019; McGee, 2021) and peers, mentors, and instructors are influenced by prevalent stereotypes about women’s STEM ability (Eddy & Brownell, 2016). Matias Dizon and colleagues (2023) also recently observed that women and Black students have stronger negative relationships than their peers between discouragement for speaking in class and GPA. Many other institutional and sociocultural contexts shape how systemic advantages contribute to grade disparities in STEM environments (Griffin, 2019; McGee, 2020).

We contend that three aspects of the current study are significant. First, this study formally introduces the SAI, showing that the selective origins of the university system still function in STEM courses today through structural inequities in course performance. Because the SAI reflects advantage in a broad sense, the index is a structural measure rather than one framed by student deficits. At the same time, the SAI (as any index) has the potential to be misused as it is not inherently anti-deficit; researchers using the SAI must actively pursue asset-based and anti-deficit framing. While we defined SAI here in terms of four categories, other dimensions of advantage (e.g., transfer status, disability status, LGBTQ + status, or English language learning status, among others) can be added and explored according to the history of a particular educational context and data availability. Indeed, we are aware of similar work at the K-12 level based on a six-factor advantage index that uses the four dimensions included here alongside English language status and disability status (Stevens, 2023), though we encourage researchers to report results with the current SAI model in future studies to support broader comparison. An example further afield, the University of California Davis has used a “disadvantage index” for more than a decade to help parse applicants to the medical school (Saul, 2023).

Second, the study is a valuable example of parallel analysis across multiple institutions; as such, the focus of the study is students’ early STEM courses writ large across research-intensive public universities. The consistent trends observed here point to systemic inequities within early STEM courses as a whole, not limited to individual instructors, courses, departments, or even institutions. Indeed, we are not aware of any work that uses data from so many large public institutions, representing various institutional contexts (e.g., land grant origin, minority-serving institution status, relative “eliteness”), to show the pervasive nature of the systemic inequities discussed herein. We note that the SAI factors included here were in part selected as most any institution would have access to similar data, facilitating further comparison. We recognize the privilege that quantitative studies have within research, as noted in dispositional tenant 5, and use this study to not only advocate for systemic change through structural and policy change (Lubienski, 2008), but also urge researchers to delve more deeply into the structural mechanisms that produce these inequities.

Third, we included courses without regard to level, which is important especially with respect to mathematics because students identifying with historically marginalized racial/ethnic groups are more likely to have introductory mathematics course placements that do not align with their aptitudes due to underestimation (Larnell, 2016). Segregation at the K-12 level also contributes to such students receiving fewer educational resources on average than white students in the U.S. (Meatto, 2019). Even when educational resources are available, discrimination from school officials has been shown to prevent racial and ethnic minorities from accessing higher-level coursework (Lewis & Diamond, 2015; Tyson, 2011). By including all course levels, our attention was focused on all early STEM courses that have traditionally played a gatekeeping role.

### Limitations

Several limitations are salient, and it is critical to grasp these as a manifestation of the choices that the research team made with respect to the study design, analyses, and interpretations. Indeed, quantitative analyses are not neutral (as stated within dispositional tenant 1) and it is imperative to understand how our choices yield limitations along different axes.

First, comparing students across universities using prescriptive administrative data (meaning data that are limited and narrow) comes at the expense of allowing individuals to articulate important factors in relation to their identities and experiences within their particular context (Lubienski & Gutiérrez, 2008). The SAI is based on the historical foundations of U.S. universities and implemented herein based on the availability of institutional data. We implemented proxies that are limited in order to make comparisons across institutions—sex rather than gender and median income by high school zip code rather than Pell grant eligibility due to lack of access to this data at two institutions. Further, the SAI does not include structural advantages related to disability, sexuality, and other elements of students’ identities because much of this data is not regularized across institutions or available for research. Higher education systems are not neutral to these identities; students experience structural advantages along these different dimensions of identity, and these are critical areas that need more research and system-level reform to strive toward equity. At the same time, though institutional data are limited, they facilitate cross-institutional analyses that can reveal broad inequities, supporting more refined future studies.

Second, the choices we made in constructing the SAI index can errantly promote binary thinking, especially with regard to race/ethnicity. Collapsing race/ethnicity into a binary category based on a structural perspective comes at the expense of seeing challenges faced by specific racial/ethnic groups, and there is the potential to misconstrue this choice as centering whiteness. These specific limitations and the implications of this approach are critical to acknowledge and discuss as the goal of this work is to highlight inequity within undergraduate STEM across institutions as a byproduct of historical structural inequities from the origins of U.S. higher education.

Finally, student groups with different identity profiles are combined herein based on their SAI number, which confounds the challenges faced by specific groups of students. It is important to reiterate that the dimensions of social identities collapsed into systemic advantages are not interchangeable, even though we are using SAI to group students by number of advantages. This also means that for SAI indices comprising multiple groups, the anomalies for some specific subsets are an underreport of their experiences given that the overall SAI results are an average of multiple groups. We collapsed the number of associated advantages that a student brings to their first STEM course to examine the manifestation of systemic inequity. In this way, the SAI is a conservative measure, yet it is able to reveal the presence of systemic inequities across institutions. We contend that the current approach is useful for describing how systemic advantages manifest across students’ intersecting backgrounds within higher education.

### Future work

To identify and change the structures and policies that enforce inequities and inequalities, future work should focus on sources of variation between institutions. Comparing outcomes in more specifically aligned courses across institutions in particular disciplines could yield exemplars of structures and systems that enable grade equity. These comparisons would help build understanding of contexts where students experience larger inequalities and where inequities do not manifest in introductory grades, pressing evermore toward equitable experiences for students in foundational STEM courses. Generalization to the introductory STEM context within other types of institutions of higher education beyond the large universities represented here is also a rich path for future studies, as is tying these analyses to qualitative data that richly cover how inequities affect students in STEM.

## Conclusion

This study adds to the robust literature on equity in STEM by showing the persistent relationship between advantage and course outcomes for students in early STEM courses. We maintain that the grade penalties observed herein reflect systemic inequities in STEM fields and show that STEM is uniquely inequitable when compared to other higher education disciplines. This study helps continue to shift conversation about student success in STEM from student-based performance differences to a metric that describes advantage broadly; clearly, the language used to address differential outcomes matters (Quinn & Desruisseaux, 2022). Addressing systemic inequity requires changing the learning environment around students. Prior research advocates for course-level changes such as working with instructors to counter ideas that students have fixed ability and intelligence (Canning et al., 2019) and fostering approaches to increase students’ growth mindset and sense of belonging (Chen et al., 2021). Importantly, within the broader pattern of inequity observed herein, campus leaders should identify how their institution contributes and work to remedy the broader systemic problems both locally and across institutions. If the goal is to support marginalized students and promote their academic excellence, we must explore how to identify but more importantly seek to correct inequities within early undergraduate STEM courses. The current study provides one approach for identifying the extent of systemic inequity present in foundational STEM courses.

### Supplementary Information

**Additional file 1: **Systemic advantage index subgroups and statistical tests for group differences.

## Data Availability

The datasets generated and/or analyzed during the current study are not publicly available due to restrictions established by the Institutional Review Boards at each institution but are available, in aggregate, from the corresponding authors upon reasonable request.

## Abbreviations

GPA: Grade point average
GPAO: Grade point average in other courses
SAI: Systemic advantage index
SEM: Standard error of the mean
STEM: Science, technology, engineering, and mathematics

## References

[CR1] Acker J (1990). Hierarchies, jobs, bodies: A theory of gendered organizations. Gender and Society.

[CR2] Adiredja AP, Louie N (2020). Untangling the web of deficit discourses in mathematics education. For the Learning of Mathematics.

[CR3] Allen WR, Jewell JO (2002). A backward glance forward: Past, present and future perspectives on historically black colleges and universities. The Review of Higher Education.

[CR4] Armstrong EA, Hamilton LT, Armstrong EM, Seeley JL (2014). “Good girls”: Gender, social class, and slut discourse on campus. Social Psychology Quarterly.

[CR5] Armstrong MA, Jovanovic J (2017). The intersectional matrix: Rethinking institutional change for URM women in STEM. Journal of Diversity in Higher Education.

[CR6] Asai DJ (2020). Race matters. Cell.

[CR7] Basile V, Black R (2019). They hated me till I was one of the “good ones”: Toward understanding and disrupting the differential racialization of undergraduate African American STEM majors. The Journal of Negro Education.

[CR8] Bauer GR, Churchill SM, Mahendran M, Walwyn C, Lizotte D, Villa-Rueda AA (2021). Intersectionality in quantitative research: A systematic review of its emergence and applications of theory and methods. SSM - Population Health.

[CR9] Bell DA (1980). Brown v. Board of Education and the interest-convergence dilemma. Harvard Law Review.

[CR10] Berkowitz SA, Traore CY, Singer DE, Atlas SJ (2015). Evaluating area-based socioeconomic status indicators for monitoring disparities within health care systems: Results from a primary care network. Health Services Research.

[CR11] Blair-Loy M, Cech EA (2022). Misconceiving merit: Paradoxes of excellence and devotion in academic science and engineering.

[CR12] Bonilla-Silva E (2006). Racism without racists: Color-blind racism and the persistence of racial inequality in the United States.

[CR13] Bourdieu P, Passeron J-C (1990). Reproduction in education, society and culture.

[CR14] Bowleg L (2008). When Black + lesbian + woman ≠ Black lesbian woman: The methodological challenges of qualitative and quantitative intersectionality research. Sex Roles.

[CR15] Brunn-Bevel RJ, Ovink SM, Byrd WC, Mahoney AD, Brunn-Bevel RJ, Ovink SM, Byrd WC (2019). Always crossing boundaries, always existing in multiple bubbles: Intersected experiences and positions on college campuses. Intersectionality in higher education: Identity and inequality on college campuses.

[CR16] Byars-Winston A, Estrada Y, Howard C, Davis D, Zalapa J (2010). Influence of social cognitive and ethnic variables on academic goals of underrepresented students in science and engineering: A multiple-groups analysis. Journal of Counseling Psychology.

[CR17] Byrd WC (2017). Poison in the ivy: Race relations and the reproduction of inequality on elite college campuses.

[CR18] Byrd WC (2021). Behind the diversity numbers: Achieving racial equity on campus.

[CR19] Byrd WC, Brunn-Bevel RJ, Ovink SM (2019). Intersectionality and higher education: Identity and inequality on college campuses.

[CR20] Canning EA, Muenks K, Green DJ, Murphy MC (2019). STEM faculty who believe ability is fixed have larger racial achievement gaps and inspire less student motivation in their classes. Science Advances.

[CR21] Carbado DW (2013). Colorblind intersectionality. Signs: Journal of Women in Culture and Society.

[CR22] Carbado DW, Harris CI (2019). Intersectionality at 30: Mapping the margins of anti-essentialism, intersectionality, and dominance theory. Harvard Law Review.

[CR23] Carnegie Classification of Institutions of Higher Education. (n.d.). *Carnegie Classification of Institutions of Higher Education.* Retrieved June 19, 2023, from https://carnegieclassifications.acenet.edu/.

[CR24] Carnevale AP, Schmidt P, Strohl J (2020). The merit myth: How our colleges favor the rich and divide America.

[CR25] Castillo, W., & Gillborn, D. (2022). How to “QuantCrit:” Practices and questions for education data researchers and users (EdWorkingPaper: 22-546). Annenberg Institute at Brown University. 10.26300/v5kh-dd65

[CR26] Castle, S., Byrd, W. C., Koester, B. P., Bonem, E., Caporale, N., Cwik, S., Denaro, K., Fiorini, S., Matz, R. L., Mead, C., Whitcomb, K. M., Singh, C., Levesque-Bristol, C., & McKay, T. A. (2021, April 9–12). *Equity in the STEM landscape: A multi-institutional approach to mapping systemic advantages within STEM courses* [Paper presentation]. American Educational Research Association (AERA) Annual Meeting, virtual. 10.3102/1689325

[CR27] Center for Research on Learning and Teaching. (n.d.). *University of Michigan Foundational Course Initiative: Basic information.* Retrieved October 13, 2023, from https://crlt.umich.edu/fci-basic-information.

[CR28] Chen S, Binning KR, Manke KJ, Brady ST, McGreevy EM, Betancur L, Limeri LB, Kaufmann N (2021). Am I a science person? A strong science identity bolsters minority students’ sense of belonging and performance in college. Personality & Social Psychology Bulletin.

[CR29] Ching CD, Roberts MT (2022). Crafting a racial equity practice in college math education. Journal of Diversity in Higher Education.

[CR30] Cho S, Crenshaw KW, McCall L (2013). Toward a field of intersectionality studies: Theory, applications, and praxis. Signs: Journal of Women in Culture and Society.

[CR31] Collins PH (2000). Gender, Black feminism, and Black political economy. The ANNALS of the American Academy of Political and Social Science.

[CR32] Collins, P. H., & Bilge, S. (2020). *Intersectionality* (2nd ed.). John Wiley & Sons.

[CR33] Costello RA, Salehi S, Ballen CJ, Burkholder E (2023). Pathways of opportunity in STEM: Comparative investigation of degree attainment across different demographic groups at a large research institution. International Journal of STEM Education.

[CR34] Covarrubias A, Vélez V, Lynn M, Dixson AD (2013). Critical race quantitative intersectionality: An anti-racist research paradigm that refuses to “let the numbers speak for themselves”. Handbook of critical race theory in education.

[CR35] Crenshaw K (1991). Mapping the margins: Intersectionality, identity politics, and violence against women of color. Stanford Law Review.

[CR36] Dancy M, Hodari AK (2023). How well-intentioned white male physicists maintain ignorance of inequity and justify inaction. International Journal of STEM Education.

[CR37] Dancy TE, Edwards KT, Earl Davis J (2018). Historically white universities and plantation politics: Anti-Blackness and higher education in the Black Lives Matter era. Urban Education.

[CR38] Davis AY (1983). Women, race & class.

[CR39] D’Ignazio C, Klein LF (2020). Data feminism.

[CR40] Dika SL, D’Amico MM (2016). Early experiences and integration in the persistence of first-generation college students in STEM and non-STEM majors. Journal of Research in Science Teaching.

[CR41] Dizon JPM, Salazar C, Kim YK, Park JJ (2023). Experiences of racial discrimination among STEM majors: The role of faculty. Journal of Student Affairs Research and Practice.

[CR42] Duran A, Dahl LS, Stipeck C, Mayhew MJ (2020). A critical quantitative analysis of students’ sense of belonging: Perspectives on race, generation status, and collegiate environments. Journal of College Student Development.

[CR43] Eddy SL, Brownell SE (2016). Beneath the numbers: A review of gender disparities in undergraduate education across science, technology, engineering, and math disciplines. Physical Review Physics Education Research.

[CR44] Engle, J., & Tinto, V. (2008). *Moving beyond access: College success for low-income, first-generation students.* Pell Institute for the Study of Opportunity in Higher Education. Retrieved February 21, 2024, from https://eric.ed.gov/?id=ED504448.

[CR45] Espinosa LL, Turk JM, Taylor M, Chessman HM (2019). Race and ethnicity in higher education: A status report.

[CR46] Farrar VS, Aguayo BYC, Caporale N (2023). Gendered performance gaps in an upper-division biology course: Academic, demographic, environmental, and affective factors. CBE—Life Sciences Education.

[CR47] Fiorini S, Tarchinski N, Pearson M, Valdivia Medinaceli M, Matz RL, Lucien J, Lee HR, Koester B, Denaro K, Caporale N, Byrd CB (2023). Major curricula as structures for disciplinary acculturation that contribute to student minoritization. Frontiers in Education.

[CR48] Garcia NM, López N, Vélez VN (2018). QuantCrit: Rectifying quantitative methods through critical race theory. Race Ethnicity and Education.

[CR49] Gillborn D, Warmington P, Demack S (2018). QuantCrit: Education, policy, ‘Big Data’ and principles for a critical race theory of statistics. Race Ethnicity and Education.

[CR50] Gin LE, Pais D, Cooper KM, Brownell SE (2022). Students with disabilities in life science undergraduate research experiences: Challenges and opportunities. CBE—Life Sciences Education.

[CR51] Griffin KA (2019). Achieving diversity at the intersection of STEM culture and campus climate.

[CR52] Griffith AL (2010). Persistence of women and minorities in STEM field majors: Is it the school that matters?. Economics of Education Review.

[CR53] Gutiérrez R, Herbel-Eisenmann B, Choppin J, Wagner D, Pimm D (2012). Context matters: How should we conceptualize equity in mathematics education?. Equity in discourse for mathematics education: Theories, practices, and policies.

[CR54] Hancock A (2007). When multiplication doesn't equal quick addition: Examining intersectionality as a research paradigm. Perspectives on Politics.

[CR55] Hancock A (2013). Empirical intersectionality: A tale of two approaches. UC Irvine Law Review.

[CR56] Harper SR (2010). An anti-deficit achievement framework for research on students of color in STEM. New Directions for Institutional Research.

[CR57] Harris J, Patton L (2018). Un/doing intersectionality through higher education research. The Journal of Higher Education.

[CR58] Haynes C, Joseph N, Patton L, Stewart S, Allen E (2020). Toward an understanding of intersectionality methodology: A 30-year literature synthesis of Black women’s experiences in higher education. Review of Educational Research.

[CR59] Hooks B (1981). Ain’t I a woman: Black women and feminism.

[CR60] Huberth M, Chen P, Tritz J, McKay TA (2015). Computer-tailored student support in introductory physics. PLoS ONE.

[CR61] Jack, A. A. (2021). The privileged poor: How elite colleges are failing disadvantaged students (2019). In *Racism in America: A reader* (pp. 170–178). Harvard University Press. 10.4159/9780674251656-020

[CR62] James NM (2023). Course letter grades and rates of D, W, F grades can introduce variability to course comparisons. Chemistry Education Research and Practice.

[CR63] Johnson E, Andrews-Larson C, Keene K, Melhuish K, Keller R, Fortune N (2020). Inquiry and gender inequity in the undergraduate mathematics classroom. Journal for Research in Mathematics Education.

[CR64] Johnston M (2018). From exclusion to integration: The N.A.A.C.P.’s legal campaign against educational segregation. Voces Novae.

[CR65] Kenyon DA, Reschovsky A (2014). Introduction to special issue on the property tax and the financing of K–12 education. Education Finance and Policy.

[CR66] King B (2015). Changing college majors: Does it happen more in STEM and do grades matter?. Journal of College Science Teaching.

[CR67] Koester BP, Grom G, McKay TA (2016). Patterns of gendered performance difference in introductory STEM courses. arXiv.

[CR68] Larnell GV (2016). More than just skill: Examining mathematics identities, racialized narratives, and remediation among black undergraduates. Journal for Research in Mathematics Education.

[CR69] Lee E (2016). Class and campus life: Managing and experiencing inequality at an elite college.

[CR70] Levinson M, Geron T, Brighouse H (2022). Conceptions of educational equity. AERA Open.

[CR71] Lewis AE, Diamond JB (2015). Despite the best intentions: How racial inequality thrives in good schools.

[CR72] Leyva LA, Quea R, Weber K, Battey D, López D (2021). Detailing racialized and gendered mechanisms of undergraduate precalculus and calculus classroom instruction. Cognition and Instruction.

[CR73] Link-Gelles R, Westreich D, Aiello AE, Shang N, Weber DJ, Holtzman C, Scherzinger K, Reingold A, Schaffner W, Harrison LH, Rosen JB, Petit S, Farley M, Thomas A, Eason J, Wigen C, Barnes M, Thomas O, Zansky S, Beall B, Moore MR (2016). Bias with respect to socioeconomic status: A closer look at zip code matching in a pneumococcal vaccine effectiveness study. SSM - Population Health.

[CR74] López N, Erwin C, Binder M, Chavez MJ (2018). Making the invisible visible: Advancing quantitative methods in higher education using critical race theory and intersectionality. Race Ethnicity and Education.

[CR75] Lorde A (1984). Sister outsider: Speeches and essays.

[CR76] Lubienski ST (2008). On gap gazing in mathematics education: The need for gaps analyses. Journal for Research in Mathematics Education.

[CR77] Lubienski ST, Gutiérrez R (2008). Bridging the gaps in perspectives on equity in mathematics education. Journal for Research in Mathematics Education.

[CR78] Malespina A, Singh C (2023). Gender gaps in grades versus grade penalties: Why grade anomalies may be more detrimental for women aspiring for careers in biological sciences. International Journal of STEM Education.

[CR79] Masta S (2019). Challenging the relationship between settler colonial ideology and higher education spaces. Berkeley Review of Education.

[CR80] Matz R, Koester B, Fiorini S, Grom G, Shepard L, Stangor C, Weiner B, Mckay T (2017). Patterns of gendered performance differences in large introductory courses at five research universities. AERA Open.

[CR81] McCammon HJ, Campbell KE, Granberg EM, Mowery C (2001). How movements win: Gendered opportunity structures and U.S. women’s suffrage movements, 1866 to 1919. American Sociological Review.

[CR82] McCoy DL, Luedke CL, Winkle-Wagner R (2017). Encouraged or weeded out: Perspectives of students of color in the STEM disciplines on faculty interactions. Journal of College Student Development.

[CR83] McGee EO (2020). Interrogating structural racism in STEM higher education. Educational Researcher.

[CR84] McGee EO (2021). Black, brown, bruised: How racialized STEM education stifles innovation.

[CR85] McGee EO, Martin DB (2011). “You would not believe what i have to go through to prove my intellectual value!” Stereotype management among academically successful Black mathematics and engineering students. American Educational Research Journal.

[CR86] Mead C, Supriya K, Zheng Y, Anbar AD, Collins JP, LePore P, Brownell SE (2020). Online biology degree program broadens access for women, first-generation to college, and low-income students, but grade disparities remain. PLoS ONE.

[CR87] Meatto, K. (2019, May 2). Still separate, still unequal: Teaching about school segregation and educational inequality. *The New York Times.* Retrieved February 21, 2024, from https://www.nytimes.com/2019/05/02/learning/lesson-plans/still-separate-still-unequal-teaching-about-school-segregation-and-educational-inequality.html.

[CR88] Michaels, K., & Milner, J. (2021). Powered by publics learning memo: The Big Ten Academic Alliance cluster exploring foundational course DFW rates, equity gaps, and progress to degree. Retrieved February 21, 2024, from https://www.aplu.org/wp-content/uploads/powered-by-publics-learning-memo-the-big-ten-academic-alliance-cluster.pdf.

[CR89] Mustaffa JB (2017). Mapping violence, naming life: A history of anti-Black oppression in the higher education system. International Journal of Qualitative Studies in Education.

[CR90] Nash MA (2019). Entangled pasts: Land-grant colleges and American Indian dispossession. History of Education Quarterly.

[CR91] National Center for Education Statistics. (n.d.). *Use the data.* Retrieved June 19, 2023, from https://nces.ed.gov/ipeds/use-the-data.

[CR92] National Center for Science and Engineering Statistics. (2021). Women, minorities, and persons with disabilities in science and engineering. National Science Foundation 21–321. Retrieved February 21, 2024, from https://ncses.nsf.gov/pubs/nsf21321.

[CR93] Office of the Assistant Secretary for Planning and Evaluation. (n.d.). *Prior HHS poverty guidelines and federal register references.* Retrieved June 19, 2023, from https://aspe.hhs.gov/topics/poverty-economic-mobility/poverty-guidelines/prior-hhs-poverty-guidelines-federal-register-references.

[CR94] Patton LD (2016). Disrupting postsecondary prose: Toward a critical race theory of higher education. Urban Education.

[CR95] Pearson MI, Castle SD, Matz RL, Koester BP, Byrd WC (2022). Integrating critical approaches into quantitative STEM equity work. CBE—Life Sciences Education.

[CR96] Posselt J (2018). Normalizing struggle: Dimensions of faculty support for doctoral students and implications for persistence and well-being. The Journal of Higher Education.

[CR97] Posselt JR (2020). Equity in science: Representation, culture, and the dynamics of change in graduate education.

[CR98] Quinn DM, Desruisseaux TM (2022). Replicating and extending effects of “achievement gap” discourse. Educational Researcher.

[CR99] R Core Team (2023). R: A language and environment for statistical computing. R Foundation for Statistical Computing, Vienna, Austria. Retrieved February 21, 2024, from https://www.R-project.org.

[CR100] Ray V (2019). A theory of racialized organizations. American Sociological Review.

[CR101] Rehrey G, Molinaro M, Groth D, Shepard L, Bennett C, Code W, Reynolds A, Squires V, Ward D, Ifenthaler D, Gibson D (2020). Supporting faculty adoption of learning analytics within the complex world of higher education. Adoption of data analytics in higher education learning and teaching.

[CR102] Reinholz DL, Matz RL, Cole R, Apkarian N (2019). STEM is not a monolith: A preliminary analysis of variations in STEM disciplinary cultures and implications for change. CBE—Life Sciences Education.

[CR103] Reinholz DL, Rasmussen C, Nardi E (2020). Time for (research on) change in mathematics departments. International Journal of Research in Undergraduate Mathematics Education.

[CR104] Reinholz DL, Ridgway SW (2021). Access needs: Centering students and disrupting ableist norms in STEM. CBE—Life Sciences Education.

[CR105] Renn KA, Reason RD (2021). College students in the United States: Characteristics, experiences, and outcomes.

[CR106] Riegle-Crumb C, King B, Irizarry Y (2019). Does STEM stand out? Examining racial/ethnic gaps in persistence across postsecondary fields. Educational Researcher.

[CR107] Russo-Tait T (2023). Science faculty conceptions of equity and their association to teaching practices. Science Education.

[CR108] Saul, S. (2023, July 2). With end of affirmative action, a push for a new tool: Adversity scores. *The New York Times.* Retrieved February 21, 2024, from https://www.nytimes.com/2023/07/02/us/affirmative-action-university-of-california-davis.html

[CR109] Seymour E, Hunter A-B (2019). Talking about leaving revisited: Persistence, relocation, and loss in undergraduate STEM education.

[CR110] Squire D, Williams BC, Tuitt F (2018). Plantation politics and neoliberal racism in higher education: A framework for reconstructing anti-racist institutions. Teachers College Record.

[CR111] Stevens, D. (2023, April 13–16). *Evaluating inequality using a six-factor index of advantage* [Conference session]*.* American Educational Research Association (AERA) Annual Meeting, Chicago, IL, United States.

[CR112] Stewart AJ, Valian V (2018). An inclusive academy: Achieving diversity and excellence.

[CR113] Stinebrickner R, Stinebrickner TR (2014). A major in science? Initial beliefs and final outcomes for college major and dropout. The Review of Economic Studies.

[CR114] Strayhorn TL (2018). College students’ sense of belonging: A key to educational success for all students.

[CR115] Thelin JR (2019). A history of American higher education.

[CR116] Tichavakunda AA (2021). Black campus life: The worlds Black students make at a historically White institution.

[CR117] Tyson K (2011). Integration interrupted: Tracking, Black students, and acting white after Brown.

[CR118] Warikoo N, Allen U (2020). A solution to multiple problems: The origins of affirmative action in higher education around the world. Studies in Higher Education.

[CR119] Whitcomb KM, Cwik S, Singh C (2021). Not all disadvantages are equal: Racial/ethnic minority students have largest disadvantage among demographic groups in both STEM and non-STEM GPA. AERA Open.

[CR120] Wilder CS (2013). Ebony and ivy: Race, slavery, and the troubled history of America’s universities.

[CR121] Witteveen D, Attewell P (2020). The STEM grading penalty: An alternative to the “leaky pipeline” hypothesis. Science Education.

[CR122] Wolbring G, Nguyen A (2023). Equity/equality, diversity and inclusion, and other EDI phrases and EDI policy frameworks: A scoping review. Trends in Higher Education.

[CR123] Wolfe P (1999). Settler colonialism.

[CR124] Wright CD, Eddy SL, Wenderoth MP, Abshire E, Blankenbiller M, Brownell SE (2016). Cognitive difficulty and format of exams predicts gender and socioeconomic gaps in exam performance of students in introductory biology courses. CBE—Life Sciences Education.

[CR125] Xie Y, Fang M, Shauman K (2015). STEM education. Annual Review of Sociology.

[CR126] Zambrana RE (2018). Toxic ivory towers: The consequences of work stress on underrepresented minority faculty.

