# Supplementary material for: Systemic advantage has a meaningful relationship with grade outcomes in students’ early STEM courses at six research universities
Source: Int J STEM Educ. 2024 Feb 23;11(1):14. doi: 10.1186/s40594-024-00474-7 (PMC10891193; doi:10.1186/s40594-024-00474-7)
Supplement: Supplementary file 1 — Additional file 1: Systemic advantage index subgroups and statistical tests for group differences. [file 40594_2024_474_MOESM1_ESM.docx]

**Table S1** Welch’s two-sample *t*-tests for differences in mean grade anomaly by systemic advantage index (SAI) group

| **Institution** | **SAI groups** | ***t*** | ***df*** | **95% *CI*** | **Institution** | **SAI groups** | ***t*** | ***df*** | **95% *CI*** |
| --- | --- | --- | --- | --- | --- | --- | --- | --- | --- |
| A | 0, 1 | -4.3 | 6420 | [-.15, -.05]*** | D | 0, 1 | -0.8 | 259 | [-.17, .07]*** |
| A | 0, 2 | -8.9 | 5022 | [-.24, -.15]*** | D | 0, 2 | -1.9 | 221 | [-.23, .00]*** |
| A | 0, 3 | -10.7 | 4452 | [-.27, -.18]*** | D | 0, 3 | -2.8 | 216 | [-.28, -.05]*** |
| A | 0, 4 | -9.7 | 5143 | [-.25, -.17]*** | D | 0, 4 | -3.3 | 217 | [-.31, -.08]*** |
| A | 1, 2 | -6.0 | 15988 | [-.12, -.06]*** | D | 1, 2 | -3.0 | 2619 | [-.11, -.02]*** |
| A | 1, 3 | -8.6 | 13650 | [-.15, -.10]*** | D | 1, 3 | -5.6 | 2164 | [-.15, -.07]*** |
| A | 1, 4 | -7.1 | 16283 | [-.14, -.08]*** | D | 1, 4 | -7.0 | 2315 | [-.18, -.10]*** |
| A | 2, 3 | -2.7 | 29770 | [-.05, -.01]*** | D | 2, 3 | -4.6 | 14653 | [-.07, -.03]*** |
| A | 2, 4 | -1.4 | 26931 | [-.04, .01]*** | D | 2, 4 | -7.0 | 15708 | [-.10, -.06]*** |
| A | 3, 4 | 1.2 | 27164 | [-.01, .04]*** | D | 3, 4 | -3.3 | 22351 | [-.05, -.01]*** |
| B | 0, 1 | -1.1 | 2105 | [-.10, .03]*** | E | 0, 1 | -0.5 | 20 | [-.59, .35]*** |
| B | 0, 2 | -4.9 | 1582 | [-.20, -.08]*** | E | 0, 2 | -1.5 | 20 | [-.81, .13]*** |
| B | 0, 3 | -8.2 | 1337 | [-.28, -.17]*** | E | 0, 3 | -1.9 | 20 | [-.90, .04]*** |
| B | 0, 4 | -11.3 | 1351 | [-.36, -.25]*** | E | 0, 4 | -2.3 | 20 | [-.99, -.05]*** |
| B | 1, 2 | -5.6 | 6031 | [-.14, -.07]*** | E | 1, 2 | -10.4 | 2216 | [-.27, -.18]*** |
| B | 1, 3 | -11.4 | 4208 | [-.22, -.16]*** | E | 1, 3 | -15.0 | 1878 | [-.35, -.27]*** |
| B | 1, 4 | -16.3 | 4320 | [-.31, -.24]*** | E | 1, 4 | -19.2 | 1944 | [-.44, -.36]*** |
| B | 2, 3 | -7.4 | 12275 | [-.11, -.06]*** | E | 2, 3 | -9.7 | 15052 | [-.10, -.07]*** |
| B | 2, 4 | -14.4 | 12710 | [-.19, -.15]*** | E | 2, 4 | -19.0 | 16815 | [-.19, -.16]*** |
| B | 3, 4 | -9.9 | 30862 | [-.10, -.07]*** | E | 3, 4 | -13.0 | 33483 | [-.10, -.08]*** |
| C | 0, 1 | -1.6 | 1927 | [-.14, .01]*** | F | 0, 1 | 0.9 | 314 | [-.08, .20]*** |
| C | 0, 2 | -6.2 | 1424 | [-.29, -.15]*** | F | 0, 2 | -0.6 | 244 | [-.17, .10]*** |
| C | 0, 3 | -8.5 | 1191 | [-.35, -.22]*** | F | 0, 3 | -0.5 | 231 | [-.17, .09]*** |
| C | 0, 4 | -8.8 | 1268 | [-.36, -.23]*** | F | 0, 4 | -2.1 | 235 | [-.27, -.01]*** |
| C | 1, 2 | -6.4 | 4080 | [-.21, -.11]*** | F | 1, 2 | -3.0 | 1777 | [-.16, -.04]*** |
| C | 1, 3 | -10.0 | 3003 | [-.27, -.18]*** | F | 1, 3 | -3.2 | 1411 | [-.16, -.04]*** |
| C | 1, 4 | -10.2 | 3399 | [-.28, -.19]*** | F | 1, 4 | -6.4 | 1522 | [-.26, -.14]*** |
| C | 2, 3 | -4.2 | 7270 | [-.10, -.04]*** | F | 2, 3 | 0.1 | 7401 | [-.03, .04]*** |
| C | 2, 4 | -4.7 | 8311 | [-.11, -.05]*** | F | 2, 4 | -5.3 | 8038 | [-.14, -.06]*** |
| C | 3, 4 | -1.0 | 13090 | [-.04, .01]*** | F | 3, 4 | -6.7 | 13000 | [-.13, -.07]*** |

*Note:* The *p* values are corrected for multiple comparisons. * *p* < .05, ** *p* < .01, *** *p* < .001.
